# Nursing education in the digital era: the role of digital competence in enhancing academic motivation and lifelong learning among nursing students

**DOI:** 10.1186/s12912-025-03199-2

**Published:** 2025-05-21

**Authors:** Shaimaa Mohamed Amin, Shimaa Abd El-fattah Mahgoub, Ahmed Farghaly Tawfik, Dalia E. Khalil, Ahmed Abdelwahab Ibrahim El-Sayed, Mohamed Hussein Ramadan Atta, Ali Albzia, Shadia Ramadan Morsy Mohamed

**Affiliations:** 1 Lecturer of Community Health Nursing, Faculty of Nursing, Damanhour University, Damanhour, Egypt; 2 Nursing Administration Department, Faculty of Nursing, Beni-Suef University, Beni-Suef, Egypt; 3 Nursing Administration Department, Faculty of Nursing, Zarqa University, Zarqa, Jordan; 4 Community Health Nursing, Faculty of Nursing, Mansoura University, Mansoura, Egypt; 5 Nursing Administration Department, Faculty of Nursing, Alexandria University, Alexandria, Egypt; 6 Nursing Department, College of Applied Medical Sciences, Prince Sattam Bin Abdulaziz University, Wadi Addawasir, Saudi Arabia; 7 Psychiatric and Mental-Health nursing Department, Faculty of Nursing, Alexandria University, Alexandria, Egypt; 8 Nursing Education Department, Faculty of Nursing, Alexandria University, Alexandria, Egypt

**Keywords:** Digital competence, Academic motivation, Lifelong learning, Nursing education, Undergraduate nursing students, Cross-sectional study

## Abstract

**Background:**

Digital competence is increasingly crucial for academic success and lifelong learning, especially in health education fields such as nursing. However, limited research examines the relationship between digital competence, academic motivation, and lifelong learning among nursing students.

**Aim:**

To assess the relationship between digital competence, academic motivation, and lifelong learning among undergraduate nursing students and explore the mediating role of academic motivation in this relationship.

**Methods:**

A descriptive comparative cross-sectional study was conducted, guided by the STROBE guidelines. Using systematic random sampling, 500 undergraduate nursing students were selected from Mansoura University, Egypt. Data were collected from July to August 2024 using three validated scales: the Students’ Digital Competence Scale, the Lifelong Learning Scale, and the Academic Motivation Scale. Descriptive statistics, Pearson correlation, and multiple regression analysis were used to analyze the data.

**Results:**

The results showed a strong positive correlation between digital competence and academic motivation (*r* = 0.53, *p* < 0.001), as well as between digital competence and lifelong learning (*r* = 0.61, *p* < 0.001). Students with higher digital competence scores also had significantly higher academic motivation (4.21 ± 0.45) and lifelong learning tendencies (4.37 ± 0.48). Multiple regression analysis confirmed that digital competence significantly predicted both academic motivation (β = 0.38, *p* < 0.001) and lifelong learning (β = 0.44, *p* < 0.001).

**Conclusion:**

Digital competence significantly enhances academic motivation and promotes lifelong learning among nursing students. The findings emphasize the need for nursing curricula to integrate digital competence training to improve educational outcomes and prepare students for future challenges in healthcare.

**Clinical trial number:**

Not applicable.

**Supplementary Information:**

The online version contains supplementary material available at 10.1186/s12912-025-03199-2.

## Introduction

The digitalization of healthcare presents opportunities and challenges for nursing education, emphasizing the need for digital competence in managing electronic health records, telemedicine platforms, and diagnostic tools [1]. Nursing students must acquire these skills early to meet modern healthcare demands and provide evidence-based, high-quality patient care. Digital competence not only enables students to navigate complex healthcare systems but also supports academic success and professional readiness [2]. As healthcare institutions increasingly adopt digital systems, equipping nursing students with technological proficiency has become essential to prepare them for evolving healthcare environments.

Despite its importance, a significant gap remains in the digital preparedness of nursing students, particularly in regions with limited access to digital resources. This lack of competence impacts academic success and lifelong professional growth, as nursing students must continuously learn throughout their careers [3]. Academic motivation, driven by intrinsic and extrinsic factors, is critical for meeting the demands of nursing programs. Students with strong digital competence are more likely to engage with their studies, excel academically, and embrace lifelong learning [4, 5]. However, limited research explores how digital competence influences academic motivation and lifelong learning in nursing education. This study addresses this gap, examining the interplay between these factors to provide insights for educators and policymakers.

## Background

### Digital competence in nursing education

Digital competence refers to efficiently using digital technologies and tools in academic and professional contexts [6]. It includes skills such as evaluating and applying information, collaborating on digital platforms, and safeguarding data in compliance with privacy regulations [7]. For nursing students, this involves utilizing digital resources like learning management systems, online databases, and simulation software, all of which are becoming integral to nursing education with the rise of e-learning and clinical digital tools [8]. These competencies are vital for academic success, as nursing programs increasingly rely on digital learning platforms and simulation-based training [9].

Digital competence also significantly impacts academic motivation. Proficient students are more likely to engage with materials, collaborate in learning activities, and leverage online resources effectively [10]. Furthermore, it fosters lifelong learning, enabling nurses to stay current with technological advancements and evidence-based practices. Leonardsen et al. (2023) [11] found that students with strong digital skills achieved higher academic success by efficiently accessing information and completing online assignments. Similarly, Ibrahim and Aldawsari (2023) [12] reported a positive link between digital competence and success in hybrid learning environments. However, many nursing students face challenges in developing these skills due to limited access to technology or training. Garzón-Artacho et al. (2021) [13] highlighted that inadequate digital competence creates barriers, particularly in online courses requiring active use of digital tools for learning and collaboration.

### Academic motivation among nursing students

Academic motivation, driven by internal and external factors, is critical for nursing students to engage in and persist through their rigorous programs and demanding clinical responsibilities [14]. Highly motivated students perform better academically and are more likely to complete their nursing education [15]. For instance, Nguyen et al. (2023) [16] found that nursing students with strong academic motivation were more engaged in classroom and clinical environments, leading to improved outcomes. This highlights the importance of motivation in shaping nursing students’ educational experiences and ensuring success in both academic and professional domains.

Digital competence significantly influences academic motivation as nursing education increasingly relies on digital tools like e-learning platforms, virtual simulations, and online collaboration technologies [17]. Proficiency in digital technologies enhances engagement and motivation by making learning more interactive and accessible [18]. Leonardsen et al. (2023) [11] reported that students with strong digital skills had higher motivation levels, particularly in courses utilizing online platforms extensively. Digital tools create a dynamic learning experience, allowing students to interact effectively with content while fostering intrinsic motivation to learn. By improving academic engagement, digital competence facilitates both academic success and an enjoyable, interactive educational process [19].

In this context, Collins (2024) found that nursing students with higher digital competence were more engaged with online learning platforms, participating in virtual discussions and collaborating on group projects [20]. This engagement fostered higher academic motivation by boosting students’ confidence in using digital tools effectively. Access to diverse resources, such as videos, quizzes, and simulations, provided flexible and interactive learning opportunities, further enhancing motivation [21]. Similarly, Haleem et al. (2022) [22] observed that students with strong digital skills frequently engaged in self-directed learning, such as reviewing online tutorials and participating in virtual simulations, which promoted intrinsic motivation by empowering students to take control of their education.

The relationship between digital competence and academic motivation is multifaceted, influencing both intrinsic and extrinsic factors [5]. Kwiatkowska and Wiśniewska-Nogaj (2022) [23] demonstrated that proficient students were intrinsically motivated to learn for personal growth and knowledge acquisition, facilitated by the ease of exploring topics using digital tools. Extrinsic motivation, driven by grades and career prospects, was also positively impacted, as students with high digital competence were more likely to complete assignments on time, achieve higher grades, and feel confident about future career success [24].

### Lifelong learning among nursing students

Lifelong learning is vital in nursing education, enabling nurses to adapt to advancements in clinical practices, technologies, and patient care protocols [25]. Students who commit to lifelong learning demonstrate better academic performance and clinical adaptability, preparing them to deliver high-quality care and respond to complex healthcare challenges [26]. Self-directed learning, a core skill in lifelong learning, empowers students to take charge of their education by identifying knowledge gaps, seeking resources, and engaging in independent study [27]. Hwang and Oh (2021) [28] found that students with strong self-directed and self-regulated learning abilities are more likely to pursue continuous professional development post-graduation, ensuring sustained competence in their nursing careers.

Reflective practice is another cornerstone of lifelong learning, as it enables students to evaluate their experiences and identify areas for improvement [29]. Regular reflection helps students adopt a growth mindset, seek additional learning opportunities, and apply new knowledge to their clinical practice [30]. Digital competence significantly impacts lifelong learning among nursing students by enhancing their ability to navigate and engage with online resources effectively [31]. Studies have shown that digitally competent students are more likely to pursue continuous education opportunities such as online courses, webinars, and virtual simulations, which extend learning beyond traditional settings [32, 33].

These digital skills enable students to access and critically evaluate new information, stay updated with the latest advancements in healthcare, and participate in virtual learning communities that foster collaboration and peer support [34]. This capability is crucial for developing a proactive learning mindset and a commitment to lifelong learning, essential for adapting to the rapidly changing demands of the healthcare field [35]. By leveraging digital tools, nursing students can continuously enhance their knowledge and skills, ensuring they remain competent and effective in their professional roles. This ongoing engagement with digital learning resources not only supports their immediate educational goals but also prepares them for future challenges in the healthcare industry [36].

### Conceptualization of study

This study is grounded in educational theories that explain how digital competence, academic motivation, and lifelong learning are interconnected. Digital competence, which encompasses the ability to use digital tools effectively for learning and problem-solving, is not only a technical skill but also a cognitive and behavioral capacity that supports active engagement with learning environments [29]. The European Framework for the Digital Competence of Educators positions digital competence as a driver of learner empowerment, autonomy, and engagement—all crucial precursors for motivation and deeper learning [37].

Academic motivation is central to self-regulated learning theories. The Expectancy-Value Theory provides a relevant framework for understanding the link between digital competence and academic motivation [38]. According to this theory, students are more likely to engage and perform well when they believe they can succeed (expectancy) and when the task is seen as valuable [39]. Digital competence enhances students’ expectancy beliefs by increasing their self-efficacy in using learning technologies. Furthermore, the ability to use digital tools meaningfully adds value to their learning process, thereby increasing academic motivation [22].

From a lifelong learning perspective, the Lifelong Learning Framework emphasizes learning to know, do, be, and live together as pillars of ongoing education [40]. Digital competence is foundational to these pillars, especially in promoting self-directed and autonomous learning [11]. The Theory of Self-Regulated Learning is also instrumental in linking academic motivation and lifelong learning [41]. Students with high motivation are more likely to set learning goals, monitor their progress, and seek resources—behaviors that align with both digital engagement and lifelong learning practices [42]. Research by Hwang & Oh (2021) [28] and Leonardsen et al. (2023) [11] supports the notion that digitally competent students are more likely to engage in reflective learning, self-assessment, and continuous development.

In this study, academic motivation is conceptualized as a mediating variable. This mediation is supported by educational models that describe how competence fuels engagement and goal-directed behavior [29]. When students feel confident in navigating digital tools, they experience a sense of mastery and are more likely to persist in learning, ultimately reinforcing their readiness to learn continuously [18]. Thus, by integrating these theoretical perspectives, this study posits that digital competence enhances academic motivation, which in turn strengthens lifelong learning tendencies among nursing students.

### Research gap

While existing studies have examined the relationship between digital competence, academic motivation, and lifelong learning, significant gaps remain in understanding the mechanisms that drive these relationships in nursing education. Prior research has largely focused on the direct association between digital competence and academic motivation [9, 11] or between digital competence and lifelong learning [13]. However, few studies have explored the mediating role of academic motivation, particularly within undergraduate nursing education, where digital technologies are becoming increasingly integrated into both classroom instruction and clinical training. By investigating this interplay relationship, this study seeks to provide deeper insights into how digital competence influences long-term learning behaviors beyond immediate academic engagement.

Moreover, much of the existing literature treats digital competence as a static skill rather than a dynamic enabler of learning processes. Studies in higher education have acknowledged that digital literacy enhances academic engagement, but they often overlook how experiential learning through digital tools fosters sustained motivation and lifelong learning [41, 42].

Furthermore, although previous studies have examined digital competence in general education settings [21, 22], limited research has focused on low-resource nursing education environments, where disparities in digital access may influence learning outcomes differently than in well-resourced institutions. Digital readiness and technology infrastructure vary widely across nursing schools, impacting students’ ability to develop digital competence, engage with academic content, and cultivate self-directed learning habits. This study contributes new insights by examining how these contextual variations shape the relationship between digital competence, motivation, and lifelong learning within a resource-constrained setting.

### Aims of the study

This study aims to assess the interplay between digital competence, academic motivation, and lifelong learning among nursing students.

## Method

### Study design

This research employed a descriptive comparative cross-sectional design and followed the guidelines set by the Strengthening the Reporting of Observational Studies in Epidemiology (STROBE).

### Setting

The study was conducted at the College of Nursing, Mansoura University, in Dakahlia Governorate, Egypt. To provide relevant context, the college integrates digital competency training into its curriculum through various courses and practical applications. The curriculum includes modules on information and communication technology (ICT) skills, electronic health records (EHR) systems, and the use of digital tools for evidence-based practice. Additionally, the college utilizes a comprehensive digital education system, including a learning management system (LMS) that facilitates online lectures, digital assignments, and interactive discussions. These systems provide students with hands-on experience in navigating digital platforms, fostering their digital competence essential for modern nursing practices.

### Sampling and study participants

The target population for this study comprises undergraduate nursing students enrolled at the Faculty of Nursing, Mansoura University, during the academic year 2023–2024. To be eligible, participants must have experience with digital-based courses, including online or hybrid learning environments incorporating digital tools and platforms. This criterion ensures a relevant digital competence assessment concerning academic motivation and lifelong learning. Participants must also be willing to participate in the study and have access to digital devices such as laptops, tablets, or smartphones, as these tools are integral to the study’s framework. Conversely, the exclusion criteria remove individuals who do not meet these requirements. Students without access to digital devices or those not engaged in online or hybrid learning environments are excluded. This approach ensures that the study’s findings accurately reflect the impact of digital tools on academic motivation and lifelong learning within the context of nursing education.

**Fig. 1:**
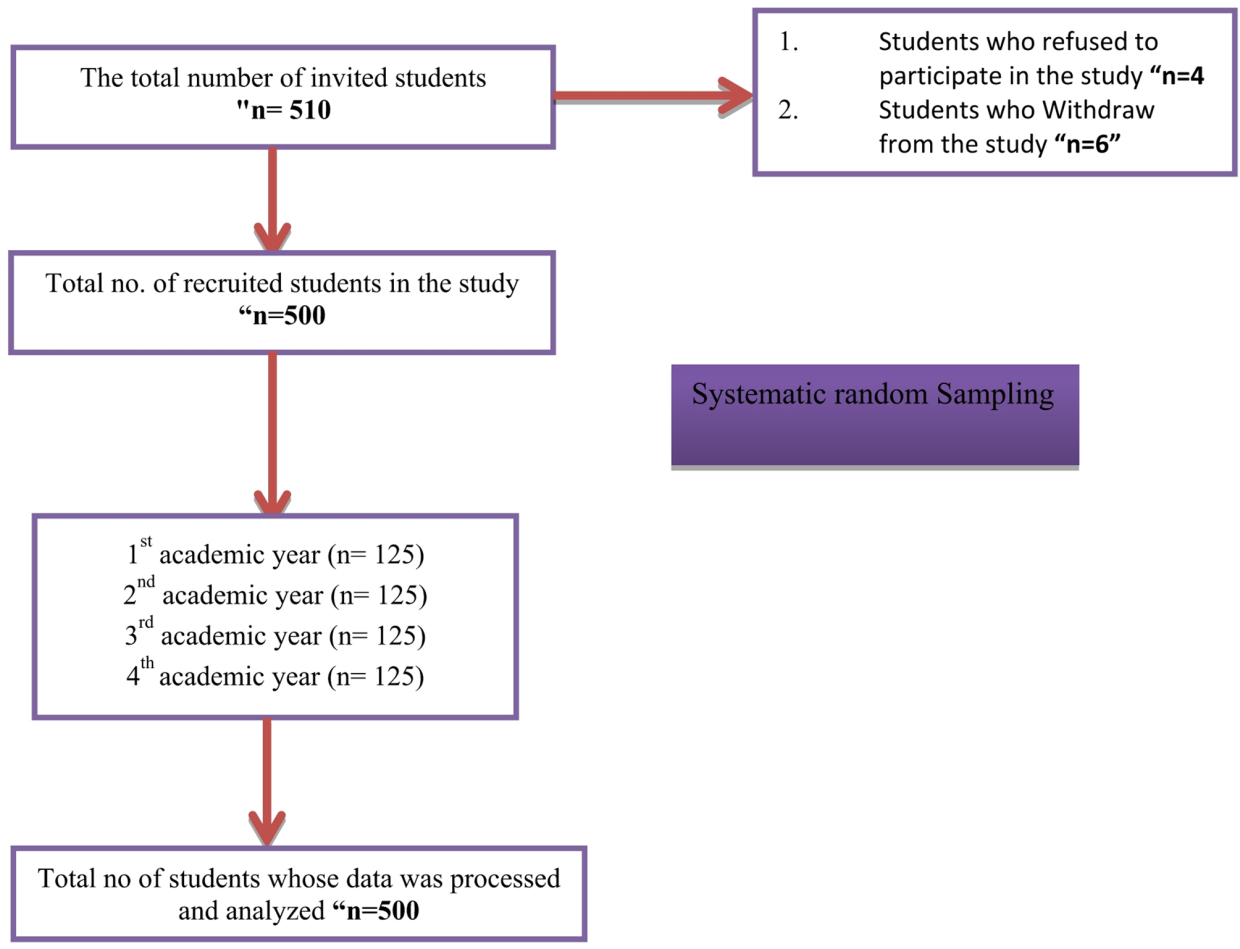
Flow chart of participants’ recruitment

The G*Power Windows 3.1.9.4 software determines the sample size required for a study assuming a multiple linear regression with a moderate effect size, an alpha of 0.05, and a power of 0.95. Considering 25 variables—including eight sociodemographic and 17 scale-related dimensions—the minimum sample size was calculated to be 495 nursing students [43]. It was upgraded to 510 to compensate for possible non-response. The final sample size for the current study, comprising participants who accepted and filled in the questionnaire, was 500. The study employed an equal allocation method to ensure representation from each academic year by selecting 125 students from each cohort. This method utilized systematic random sampling, which involves selecting every 30th student from a list or population in a predetermined order, as shown in Figure 1.

### Measurements of interest

#### Demographic form

The demographic form included data on participants’ age, sex, occupation, place of residence, family type, family income, and parent’s level of education. Additionally, to better contextualize the relationships between these general characteristics and key study variables, cultural and national differences were considered. Given the study’s setting in Egypt, cultural factors such as social norms, access to technology, and educational practices were taken into account, as these can significantly influence digital competency and academic motivation. These contextual elements provide a more nuanced understanding of the participants’ experiences and how they relate to the variables under investigation.

### Students’ digital competence scale

The scale, developed by Tzafilkou et al. (2022) [44] to evaluate the digital competence of higher education students, consists of 28 items divided into six subscales: Search, Find, Access (5 items); Develop, Apply, Modify (6 items); Communicate, Collaborate, Share (3 items); Store, Manage, Delete (5 items); Evaluate (6 items); and Protect (3 items). Each item is rated on a five-point Likert scale ranging from strongly disagree [1] to strongly agree [5], with higher scores indicating a higher level of digital competence. The scale’s internal consistency was confirmed through high factor loadings (> 0.5) and Cronbach’s alpha values, demonstrating the reliability of the measurement items and constructs.

Validity was established using Confirmatory Factor Analysis (CFA), which showed a good model fit with values of NFI (0.667), Chi-Square (843.442), and RMSEA (0.088), indicating that the constructs accurately measured the intended variables. Cronbach’s alpha of the present study was 0.88. After translating the scale into Arabic, content validity was assessed through exploratory factor analysis, which revealed satisfactory loadings before and after varimax rotation. These factors collectively accounted for 78.151% of the total variance, confirming the robust construct validity of the scale. Additionally, the Kaiser-Meyer-Olkin measure and Bartlett’s sphericity test supported the data’s suitability for factor analysis, further endorsing the scale’s validity for Arabic-speaking populations.

### The Academic Motivation Scale (AMS-C-28)

Developed by Vallerand et al. (1992) [46], the Academic Motivation Scale (AMS) assesses various types of motivation within an educational context. It includes 28 items across seven subscales: Intrinsic Motivation to Know, Intrinsic Motivation towards Accomplishment, Intrinsic Motivation to Experience Stimulation, Extrinsic Motivation Identified, Extrinsic Motivation Introjected, Extrinsic Motivation External Regulation, and Amotivation. Each subscale consists of four items rated on a five-point Likert scale ranging from “Not at all” [1] to “Exactly” [5]. Higher intrinsic or extrinsic motivation scores indicate stronger motivation for academic activities, while higher a motivation scores reflect a lack of motivation. The AMS demonstrates robust psychometric properties, including solid reliability and validity. Reliability is supported by high internal consistency, with Cronbach’s alpha values ranging from 0.70 to 0.90 for the subscales and good test-retest reliability.

Validity is confirmed by confirmatory factor analysis, which validates the scale’s three-factor structure. Recent research by Fatima et al. (2021) [47] highlights the scale’s excellent psychometric properties. Cronbach’s alpha of the current study was 0.88. Following translation into Arabic, content validity was established through exploratory factor analysis, which revealed satisfactory loadings before and after varimax rotation, accounting for 78.151% of the total variance. The Kaiser-Meyer-Olkin measure and Bartlett’s test of sphericity further supported the scale’s validity for Arabic-speaking populations.

### Lifelong learning scale

The Lifelong Learning Scale, developed by Wielkiewicz and Meuwissen (2014) [45], evaluates positive behaviors and attitudes related to learning, curiosity, and critical thinking. It comprises 16 items rated on a five-point Likert scale from never [1] to always [5], with higher scores reflecting a stronger inclination toward lifelong learning, including more significant curiosity, ongoing motivation, and practical application of learning. The scale demonstrated robust reliability, with a Cronbach’s alpha of 0.91. Significant correlations with academic performance, personality traits, and relevant behaviors reinforced construct validity. It successfully differentiated between groups based on study abroad experience, academic major, and demographic variables. Cronbach’s alpha of the present study was 0.98. After translating the scale into Arabic, content validity was affirmed through exploratory factor analysis, showing satisfactory loadings before and after varimax rotation, accounting for 80.345% of the total variance. Additionally, the Kaiser-Meyer-Olkin measure and Bartlett’s test of sphericity supported the scale’s validity for Arabic-speaking populations.

### Study procedures

#### Tool preparation and pilot study

The research instruments were carefully translated into Arabic, including the Students’ Digital Competence Scale, the Lifelong Learning Scale, and the Academic Motivation Scale (AMS-C-28). Bilingual experts fluent in English and Arabic translated to ensure accuracy and cultural appropriateness. To verify the translations, a back-translation into English was performed to ensure linguistic equivalence and resolve any discrepancies. After translation and back-translation, face validity was assessed by having expert panels review the instruments to confirm that they accurately reflected the intended constructs within the Arabic context. Additionally, feedback was collected from potential participants to ensure the clarity, relevance, and cultural suitability of the translated items. Reliability was evaluated using statistical methods, including Cronbach’s alpha, to confirm internal consistency. A pilot study with 50 students was conducted to test the instruments’ clarity, relevance, and reliability, with these participants excluded from the main study. The pilot study results indicated that no modifications were needed, confirming the instruments’ suitability for the primary research.

### Data collection

Data collection for this study took place from July to August 2024. It began after securing the necessary permissions and obtaining Excel spreadsheets with the details of all undergraduate students from the academic affairs department. Participants were selected using a random number generator, repeated until the required number of students from each academic year was achieved. Before data collection, researchers explained the study’s objectives to each student, emphasizing voluntary participation. Written informed consent was obtained from all participants as a prerequisite for their involvement. Researchers assured participants that their responses would remain confidential to ensure confidentiality and build trust. Questionnaires were distributed in quiet locations, such as empty lecture halls and libraries, between 9 am and 2 pm from Saturday to Thursday. On average, participants spent 15 to 20 min completing each questionnaire.

### Ethical considerations

Ethical approval for the study was granted by the Research Ethics Committee of the Faculty of Nursing at Mansoura University, Egypt (Serial No: 103-C). The study adhered to all applicable regulations, local laws, and the ethical principles outlined in the Declaration of Helsinki. Participation was entirely voluntary, and participants were fully informed of the study’s objectives. Anonymity was ensured, as no identifying information was collected, and responses could not be traced back to any individual. Written informed consent was obtained from all participants prior to data collection.

### Statistical analysis

Before being entered into the computer, data were thoroughly verified. The Statistical Package for the Social Sciences (SPSS version 25.0) was utilized for this purpose, followed by the analysis and tabulation of the data. Descriptive statistics were employed to illustrate the characteristics and variables of the study participants. A significant result was noted when *p* ≤ 0.001, with a significance threshold set at *p* < 0.05. A path analysis model was created to examine mediation using SPSS-AMOS version 26. The hypothesized model was assessed using AMOS’s structural equation modeling (SEM). The confirmatory factor analyses (CFA) findings indicated that the model fit the data well. The tests applied in this study were the chi-square (χ2) test of model fit, with a ratio of χ2/df < 5 representing a good model fit. Other tests used were the comparative fit index (CFI ≥ 0.95), and root mean square error of approximation (RMSEA < 0.08) [51]. The data showed a normal distribution according to the Kolmogorov-Smirnov test, so parametric tests were applied. The multicollinearity of the variables was assessed, with a confirmed tolerance greater than 0.1 and a variance inflation factor (VIF) below 3, indicating no multicollinearity. Pearson’s correlation was employed to assess the bivariate correlations between the variables, including the Students’ Digital Competence, Lifelong Learning, Academic Motivation, and their sub-dimensions and Spearman’s rank correlation for ordinal categorical variables (e.g., Academic Year).

## Results

### Characteristics of study participants

The study included 500 nursing students, with 63% aged between 20 and 22 years and a female majority of 54.2%. Most participants (73.2%) resided in rural areas, while 60% reported sufficient family income, and 12.8% indicated it was insufficient. Parental education levels revealed 47.4% of mothers and 41.6% of fathers had completed secondary education, with 28% and 34%, respectively, having university-level education.

Regarding technology, 57.8% of students reported having access to technology, while 42.2% did not. Internet access was available at home for 45%, through university/college for 21%, and 26.2% relied on mobile data; 7.8% reported no access. E-learning was used by 41.2%, hybrid learning by 29.8%, and traditional lectures by 29%. Simulation-based learning was the most common clinical teaching method (43%), followed by problem-based learning (30.8%) and field trips (21.6%). Digital experience varied, with 42.4% reporting advanced skills in online platforms and 32% as beginners. For digital tools, 43.4% had no experience, while 28.4% had more than three years of experience, See supplementary file Table 1S.

**Table 1:** Correlations between study variables and study participants’ characteristics (*n* = 500)

Participants’ characteristics	Digital Competence	Academic motivation	Lifelong Learning
Age	0.042	0.073	0.007
Academic year	**0.124** ^**^	**0.094** ^*^	**0.108** ^*^
Occupation	**0.106** ^*^	0.086	**0.102** ^*^
Place of residence	0.003	-0.063	-0.048
Family type	0.005	0.044	-0.044
Family income	-0.019	-0.040	0.006
Mother’s educational level	0.053	0.021	0.049
Father’s Educational Level	**0.099** ^*^	0.054	0.077
Technology Access	**0.141** ^**^	0.085	0.042
Internet Access	**0.171** ^**^	**0.113** ^*^	**0.121** ^**^
Teaching method	**0.147** ^**^	**0.099** ^*^	**0.088** ^*^
Clinical Teaching method	**0.109** ^*^	0.069	**0.132** ^**^
Experience with online learning platforms	**0.154** ^**^	**0.115** ^*^	**0.131** ^**^
Experience with digital tools	0.213**	0.196**	0.199**

**. Correlation is significant at the 0.01 level (2-tailed). *. Correlation is significant at the 0.05 level (2-tailed)

Table 1 displays the correlations between participants’ characteristics and key study variables digital competence, academic motivation, and lifelong learning—for a sample of 500 individuals. The table highlights a mix of significant and non-significant relationships. Academic year displays substantial correlations with all study variables: digital competence, academic motivation, and lifelong learning r = (0.124, 0.094, & 0,108 respectively), occupation correlated with digital competence and lifelong learning r = (0.106, & 0.102 respectively). Father’s Educational Level correlated with digital competence *r* = 0.99.

**Table 2:** Path analysis of direct and indirect effects of digital competence on lifelong learning mediated by academic motivation

Variable 1	Direction	Variable2	β	S.E.	C.*R*.	*P*	Significance
A.M.	<---	D.C	0.671	0.035	11.569	***	significant
Search	<---	D.C	0.829				
Communicate	<---	D.C	0.902	0.026	25.957	***	significant
Store	<---	D.C	0.845	0.049	23.334	***	significant
Evaluate	<---	D.C	0.880	0.052	24.901	***	significant
Protect	<---	D.C	0.857	0.028	23.854	***	significant
A motivation	<---	A.M.	0.596				significant
EM. Regulation	<---	A.M.	0.858	0.100	14.826	***	significant
EM. Introjected	<---	A.M.	0.893	0.104	15.192	***	significant
EM. Identified	<---	A.M.	0.911	0.105	15.369	***	significant
IM. Experience	<---	A.M.	0.896	0.104	15.226	***	significant
IM. Accomplishment	<---	A.M.	0.886	0.107	15.117	***	significant
IM. Know	<---	A.M.	0.870	0.101	14.953	***	significant
Develop	<---	D.C	0.849	0.051	23.482	***	significant
Lifelong Learning	<---	D.C	0.321	0.159	7.973	***	significant
Lifelong Learning	<---	A.M.	0.554	0.335	10.748	***	significant

**β**: Standardized Coefficients, **S.E.**: standard error, **C.R**.: Critical ratios, *: Statistically significant at *p* ≤ 0.05

D.C = digital competence, A.M = academic motivation, E.M = extrinsic motivation, I.M = intrinsic motivation

**Fig. 2:**
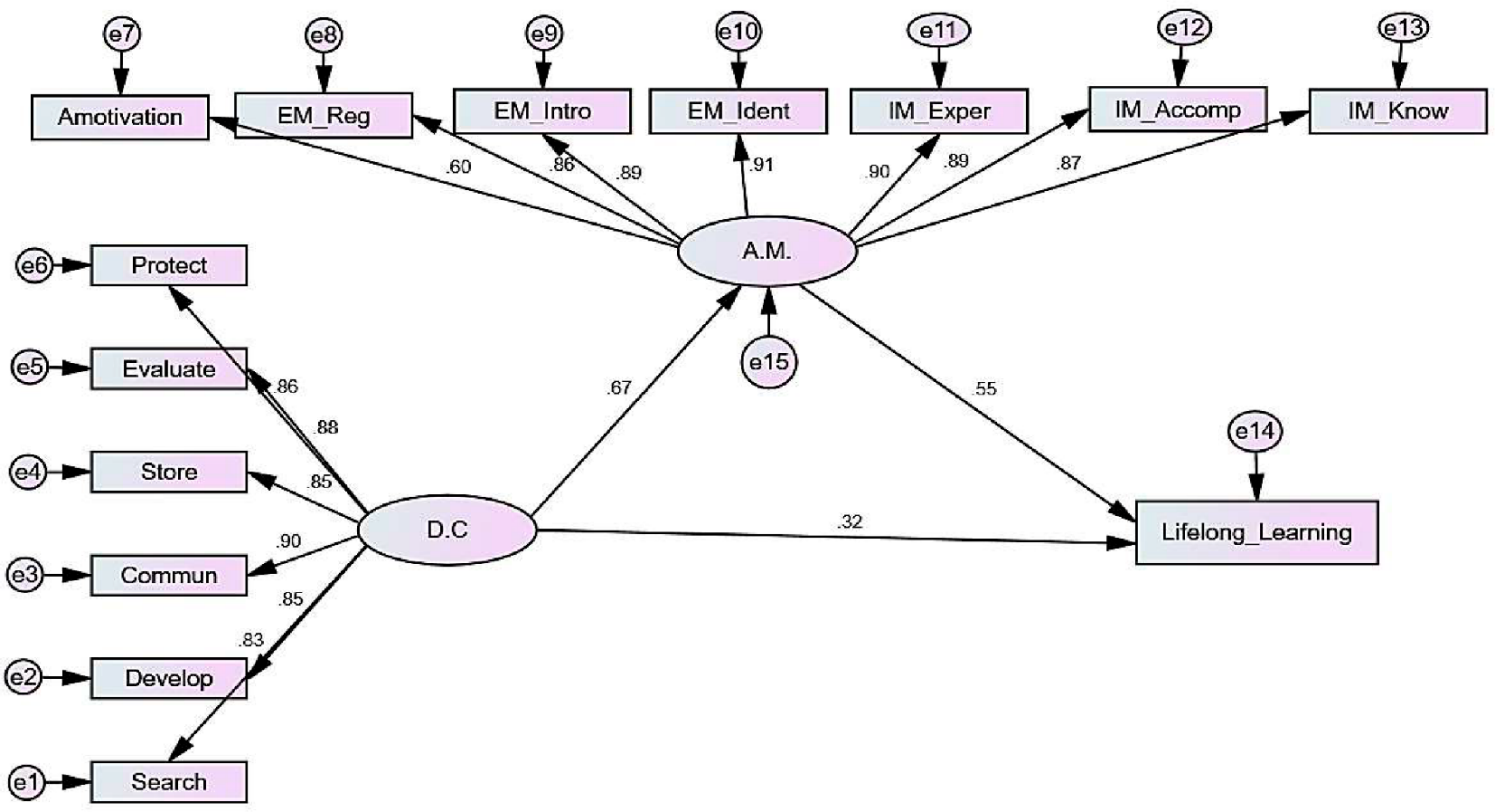
Path analysis of direct and indirect effects of digital competence on lifelong learning mediated by academic motivation. Model fit parameters X^2^/DF, GFI; CFI; RMSEA (4.41, 0.905, 0,963, 0.08). GFI = Goodness of Fit Index, CFI = Comparative fit index, and RMSEA = Root Mean Square Error of Approximation. Model Chi square = 330.47, DF = 75, *p* < 0.000*. D.C = digital competence, A.M = academic motivation, IM_Know = Intrinsic Motivation to Know, IM_Accomp = Intrinsic Motivation towards Accomplishment, IM_Exper = Intrinsic Motivation to Experience Stimulation, EM_Ident = Extrinsic Motivation Identified, EM_Intro = Extrinsic Motivation Introjected, EM_Reg = Extrinsic Motivation External Regulation

Table 2; Fig. 2. present the results from the path analysis, illustrating standardized regression weights, standard errors (SE), critical ratios (CR), and significance (p-values) of both the direct and indirect effects of digital competence on Lifelong Learning. The statistical analysis was conducted using SPSS-AMOS. The study’s variables produced reliable estimates, with the comparative fit index indicating a high model fit and the root mean square approximation error also indicating a good fit. The model fit parameters met satisfactory standards (X^2^/DF, GFI; CFI; RMSEA “4.41, 0.905, 0,963, 0.08”). The results of the path analysis presented show a significant impact of both digital competence and academic motivation on lifelong learning. The coefficients (β) for digital competence and academic motivation are both statistically significant (*p* < 0.001), indicating a strong positive effect on lifelong learning. These effects are partially mediated through academic motivation as the direct effect.

Table 3 presents the results of a hierarchical multiple linear regression analysis exploring the relationship between digital competence, academic motivation, various study participant characteristics, and lifelong learning (*n* = 500). Model 1, which includes only digital competence and academic motivation, explains 61.6% of the variance in lifelong learning (R-squared = 0.616) and demonstrates a statistically significant overall fit (F = 398.5, *p* < 0.000). Both digital competence (B = 0.227, *p* < 0.000) and academic motivation (B = 0.366, *p* < 0.000) show positive and significant relationships with lifelong learning.

Model 2 expands upon Model 1 by adding age, academic year, occupation, place of residence, family type, family income, parental education levels, technology access, internet access, teaching method, clinical teaching method, experience with online learning platforms, and experience with digital tools. While Model 2 explains slightly more variance in lifelong learning (R-squared = 0.631), the increase of 0.015 is not statistically significant (F change = 1.37, *p* = 0.163).

Furthermore, many of the added variables in Model 2 are not individually statistically significant, with only family type (B=-2.232, *p* = 0.017) and clinical teaching method (B=-0.944, *p* = 0.027) showing significant relationships with lifelong learning. Digital competence (B = 0.220, *p* < 0.000) and academic motivation (B = 0.369, *p* < 0.000) remain significant predictors in Model 2, with coefficients very similar to those in Model 1. This suggests that the additional variables in Model 2 do not substantially confound the relationships between digital competence, academic motivation, and lifelong learning. In conclusion, while Model 2 provides a more comprehensive picture, the lack of significant improvement in explanatory power and the non-significance of many added variables suggest that the more parsimonious Model 1 might be preferred.

**Table 3:** Regression analysis of relationships between digital competence, academic motivation & study participants’ characteristics and their Life-long learning (*n* = 500)

Model		Unstandardized coefficients		Standardized coefficients	t	Sig.
		B	Std. Error	Beta		
1	(Constant)	5.975	1.840		3.248	0.001
	Digital Competence	0.227	0.023	0.355	10.067	0.000
	Academic Motivation	0.366	0.025	0.515	14.605	0.000
2	(Constant)	11.822	7.092		1.667	0.096
	Digital Competence	0.220	0.023	0.344	9.623	0.000
	Academic Motivation	0.369	0.025	0.520	14.643	0.000
	Age	-1.104	0.931	-0.033	-1.187	0.236
	Academic year	0.926	1.436	0.019	0.645	0.519
	Occupation	1.177	1.126	0.030	1.045	0.297
	Place of residence	-0.921	1.071	-0.025	-0.860	0.390
	Family type	-2.232	0.929	-0.068	-2.404	0.017
	Family income	0.595	0.652	0.026	0.913	0.362
	Mother’s educational level	-0.061	0.722	-0.003	-0.084	0.933
	Father’s Educational Level	0.221	0.696	0.012	0.317	0.751
	Technology Access	-0.627	0.932	-0.019	-0.672	0.502
	Internet Access	0.343	0.413	0.023	0.829	0.408
	Teaching method	-0.676	0.574	-0.033	-1.178	0.239
	Clinical Teaching method	-0.944	0.425	-0.062	-2.222	0.027
	Experience with online learning platforms	-0.611	0.578	-0.030	-1.057	0.291
	Experience with digital tools	-0.200	0.422	-0.013	-0.475	0.635

a. Dependent Variable: Lifelong Learning

Model1, F = 398.5, *P* > 0.000, R square = 0.616

Model2, F = 51.5, *P* > 0.000, R square = 0.631, R square change = 0.015, F Change = 1.37, *p* = 0.163

## Discussion

In the rapidly evolving digital era, digital competence has become essential for academic success and lifelong learning, particularly in health education fields like nursing. However, research exploring the interplay between digital competence, academic motivation, and lifelong learning among nursing students remains limited. This study aimed to assess these relationships and examine the mediating role of academic motivation. The findings revealed significant positive correlations between digital competence and both academic motivation and lifelong learning. Additionally, digital competence was a strong predictor of both variables, highlighting the importance of integrating digital competence training into nursing education to enhance students’ learning experiences and future professional preparedness.

### Level of digital competence, academic motivation, and lifelong learning

Our findings revealed that nursing students had average digital competence, academic motivation, and lifelong learning. Similarly, a study by Leonardsen et al., 2023 [11] showed that 236 nursing students expressed positive attitudes toward using digital technology. Regarding academic motivation, our findings align with those of El-Sayed and Abdelaliem (2023), who reported strong academic motivation among nursing internship students in Egypt [14]. This consistency suggests that Egyptian nursing students, like their counterparts in other regions, are highly motivated in their academic pursuits. However, additional comparative studies are needed to determine whether similar motivation levels are observed among nursing students from different countries.

Additionally, our results indicated that nursing students possess considerable lifelong learning abilities, which is consistent with findings from Zhao et al. (2021), who highlighted the importance of digital competence in higher education research [21]. These findings suggest that the commitment to lifelong learning among nursing students may be a global trend, though further cross-national comparisons are warranted to explore potential differences.

### The interplay between study variables

Our study in Egypt found a positive association between digital competence and academic motivation among nursing students, indicating that those with higher proficiency in digital technologies are more motivated to succeed academically. This aligns with findings from Ibrahim and Aldawsari (2023) in Saudi Arabia, who reported that nursing students’ readiness for e-learning was linked to their satisfaction and motivation [12]. This suggests that across different countries, digital skills play a crucial role in shaping students’ motivation for academic success.

However, cultural and national factors may influence this relationship. In our Egyptian context, rural residency and limited access to technology present challenges that can impact both digital competence and academic motivation. These disparities highlight the need for targeted interventions to bridge gaps in digital competence, particularly in resource-constrained settings. Similarly, a study conducted in Egypt by Amin et al. (2024) emphasized that attitudes toward digital transformation in healthcare vary, which could reflect broader differences in digital readiness across regions [18].

Digital skills enable students to access various educational resources, enhance learning efficiency, and streamline communication with peers and instructors. As digital tools become increasingly integrated into academic environments, students with higher digital competence may feel more confident and capable, which in turn boosts their academic motivation. This finding aligns with Haleem et al. (2022), who highlighted the transformative role of digital technologies in education, evolving from simple knowledge providers to co-creators of information, mentors, and assessors [22].

Moreover, the integration of digital tools, such as software for presentations and project development, has simplified academic tasks, making learning more engaging and accessible. This ease of use reduces physical burdens, like carrying heavy books or notebooks, and enhances students’ interest in research and learning. Furthermore, digital competence contributes to better time management, personalized learning experiences, and access to online platforms that foster collaboration and innovation factors that further enhance academic motivation. Consistent with our findings, Galindo-Domínguez and Bezanilla (2021) in Spain found that time management and academic self-efficacy mediate the relationship between digital competence and academic success [34].

The significant correlations between experience with online learning platforms and all study variables of digital competence, academic motivation, and lifelong learning further reinforce the positive association between digital competence and academic motivation among nursing students in Egypt. Online learning platforms require students to develop and apply digital skills, enhancing their technological proficiency. Simultaneously, these platforms foster academic motivation by providing engaging, flexible, and interactive learning environments tailored to individual needs.

Similar findings have been reported in other contexts. For example, Ali et al. (2024) in Egypt found that nursing students’ use of online platforms was linked to improved time management, which is a key factor in academic success [36]. Likewise, Rushdan et al. (2024) highlighted that different study modalities influence nursing students’ engagement and academic self-concept in Egypt, further emphasizing the role of digital competence in shaping their learning experiences [48]. These findings suggest that, across various educational settings, the ability to navigate online learning platforms can lead to greater confidence in digital abilities and a more proactive approach to learning, reinforcing the interconnection between digital competence and academic motivation.

Moreover, our findings revealed a positive relationship between digital competence and lifelong learning among nursing students. This aligns with the study by Garzón-Artacho et al. (2021) in Spain, which emphasized that from the teacher’s perspective, digital competency represents a major educational challenge that the academic community must address [13]. The study highlights the need for educators to adapt their teaching practices innovatively and align them with the technological advancements shaping modern education.

This relationship underscores the importance of digital skills in promoting constant education and personal development. In today’s fast-paced, technology-driven world, navigating digital tools and platforms is essential for accessing information, engaging in online courses, and participating in professional development opportunities. Individuals with solid digital competencies are often more adaptable and open to new learning experiences, enabling them to pursue knowledge beyond traditional educational settings. This relationship highlights that digital competence enhances immediate learning outcomes and equips individuals with the skills necessary for lifelong learning, allowing them to stay relevant in their fields, adapt to changes, and engage in self-directed learning.

Furthermore, the significant correlations between the teaching method and all study variables, digital competence, academic motivation, and lifelong learning, reinforce the positive association between digital competence and academic motivation among nursing students. Effective teaching methods, particularly those incorporating interactive, technology-enhanced, and student-centered approaches, can foster digital competence and motivate students academically. For instance, teaching strategies integrating digital tools and real-world applications may enhance students’ ability to use technology effectively while increasing their interest and engagement with the learning material. This dual benefit underscores the role of well-designed educational approaches in promoting both the technical and motivational aspects of nursing education, ultimately contributing to students’ preparedness for lifelong learning and professional practice.

The positive relationship between academic engagement and lifelong learning among nursing students in Egypt aligns with findings from other countries, highlighting the global significance of this connection. Our results indicate that individuals actively engaged in educational pursuits tend to develop strong critical thinking, problem-solving, and self-directed learning skills, all of which are essential for lifelong learning. Academic environments foster curiosity and a desire for knowledge, encouraging students to continue learning beyond formal education.

Similarly, Chukwuedo et al. (2021) in Nigeria found that self-directed learning plays a crucial role in motivating academic engagement and lifelong learning among vocational and adult education students [25]. This suggests that fostering autonomy in learning can enhance students’ ability to acquire and apply knowledge across different educational contexts. Likewise, Hwang and Oh (2021) in Korea proposed that integrating academic self-efficacy and self-regulated learning is an effective approach to implementing self-directed learning, which enhances nursing students’ problem-solving skills [28]. Their study underscores the importance of personal motivation and self-discipline in achieving long-term learning goals.

Additionally, academic success often builds confidence in one’s ability to acquire new skills and adapt to changing information, making lifelong learning an appealing and attainable goal [25, 38]. Furthermore, those who experience academic fulfillment are more likely to recognize the value of continuous learning in personal growth and professional advancement, leading them to seek new learning opportunities.

The significant correlations between academic year and the study variables digital competence, academic motivation, and lifelong learning suggest that as nursing students progress through their educational years, there is a gradual improvement in these areas. Increased digital competence could result from repeated interactions with digital platforms, technologies, and electronic health records, integral to modern nursing education. Advanced coursework and clinical training likely necessitate more frequent and sophisticated use of digital tools, enhancing students’ technological proficiency (Amin et al., 2024) [36].

The positive correlation with academic motivation might stem from a growing sense of purpose and confidence as students advance toward completing their degree. Senior students may become more motivated due to their proximity to graduation and the opportunities for professional practice that follow. As students gain more exposure to clinical practice and theoretical knowledge, they likely recognize the need to stay updated in a rapidly evolving healthcare environment.

The study highlights both the direct and indirect effects of digital competence on lifelong learning, with academic motivation serving as a key mediator. Our findings in Egypt align with global research, reinforcing the crucial role of digital skills in shaping students’ learning behaviors. Directly, digital competence enhances lifelong learning by providing better access to online educational resources, tools, and platforms, enabling students to engage more effectively with learning materials. Additionally, it fosters self-directed learning, empowering individuals to take greater control of their education. Similarly, Mehrvarz et al. (2021) in Iran found that digital informal learning mediates the relationship between students’ digital competence and academic performance, indicating that engaging with technology enhances students’ ability to acquire and apply knowledge [1].

The indirect effect of digital competence on lifelong learning is facilitated by increased academic motivation. Interactive and technology-driven learning environments help make education more engaging and relevant, motivating students to continue learning. In support of this, Pan et al. (2024) in China reported that digitalization in higher education institutions positively moderates the relationship between students’ personality traits and learning behaviors, demonstrating that access to digital tools enhances student engagement [3].

Moreover, the perception of digital skills as critical for both personal and professional growth further strengthens students’ motivation to engage in lifelong learning. Koskinen et al. (2024) emphasized that methodological approaches integrating digital competence, such as virtual reality simulations, can enhance nursing students’ educational experiences and skill acquisition [50].

Our results indicate that technology access, internet access, teaching methods, clinical teaching methods, experience with online learning platforms, and experience with digital tools positively correlate with digital competence, academic motivation, and lifelong learning. These elements represent various forms of digital formation that significantly enhance digital competence. By providing learners with access to technology and internet resources, along with practical teaching methodologies and hands-on experience with online platforms, individuals are better equipped to develop their digital skills. This development fosters greater academic motivation and supports digital competence’s direct and indirect effects on lifelong learning [51, 52].

As learners become more adept at navigating digital environments, they are more likely to engage in self-directed learning, remain motivated in their educational pursuits, and ultimately commit to lifelong learning. Thus, these aspects of digital formation play a crucial role in facilitating the interplay between digital competence and lifelong learning, mediated by academic motivation.

### Strengths and limitation

This study employed robust statistical analysis techniques, including path analysis and regression modeling, which provided reliable estimates of the relationships among digital competence, academic motivation, and lifelong learning. It addresses a critical area of interest in education by emphasizing the pivotal roles of digital competence and academic motivation in fostering lifelong learning. The findings offer practical implications for educators and policymakers, highlighting strategies to enhance lifelong learning through targeted interventions. Additionally, the comprehensive measurement of digital competence and academic motivation enhances the reliability of the results, offering a nuanced understanding of their roles in the learning process.

However, some limitations should be acknowledged. First, the study was conducted at a single site, which may limit the generalizability of the findings. The participants’ specific academic and cultural context may not fully represent other educational settings. Future research could address this limitation by conducting multi-site studies to capture diverse contexts and improve external validity. Second, the study relied on voluntary participation, introducing the possibility of response bias. Participants may have provided socially desirable responses, particularly regarding their digital competence and academic motivation, which could affect the accuracy of the self-reported data. Future studies could consider employing anonymous surveys or alternative data collection methods, such as implicit measures or triangulation with objective data, to reduce this bias. Finally, the study relied solely on self-reported data, which, while helpful in capturing participants’ perceptions, is subject to recall errors and potential inaccuracies. Future research could strengthen the validity of findings by incorporating objective measures, such as digital performance metrics, observational data, or peer evaluations, to complement self-reported data and provide a more holistic view of the constructs examined.

### Implications

Based on the study’s findings, educational institutions should prioritize the integration of digital competence into nursing curricula to enhance academic motivation and lifelong learning. A structured, cross-disciplinary approach is essential to embedding digital competence across various courses, ensuring students develop and apply these skills in diverse academic and clinical settings. Integrating digital competence into nursing education can be achieved through well-designed assignments, interactive projects, and technology-enhanced learning activities.

However, incorporating digital competence into curricula presents challenges, including varying levels of digital competence among educators and students, limited institutional resources, and unequal access to technology. To address these challenges, institutions must ensure equitable access to digital tools and infrastructure. Strategies such as faculty training programs, peer mentoring, and structured technology support systems can help bridge digital competence gaps among both educators and students.

To enhance student engagement and motivation, educational institutions should adopt innovative pedagogical approaches that integrate digital tools into active learning. Techniques such as project-based learning flipped classrooms, and gamification can increase student engagement, promote self-directed learning, and reinforce the relevance of digital skills in clinical practice. Real-life case studies, problem-solving exercises, and simulation-based training can further strengthen students’ motivation by making learning experiences more practical and aligned with their professional aspirations. Additionally, incorporating formative feedback mechanisms and recognizing student achievements can enhance their self-efficacy and intrinsic motivation in digital learning environments.

The study also underscores the importance of lifelong learning in nursing education. To foster continuous professional growth, institutions should provide technology-enhanced learning environments, including online platforms, virtual labs, and interactive simulations. These tools can enable students to learn at their own pace, collaborate with peers, and develop essential digital competencies needed for modern healthcare settings.

Finally, ongoing professional development for educators is crucial in ensuring the effective integration of digital competence into teaching practices. Training programs should equip faculty with the skills to use learning management systems (LMS), digital content creation tools, and data-driven assessment methods to enhance student learning outcomes. Establishing communities of practice, where educators share best practices and experiences, can further support faculty in adapting to digital advancements in nursing education.

By addressing these institutional and pedagogical challenges, nursing education can better prepare students for the demands of an increasingly digital healthcare landscape, fostering academic motivation, self-efficacy, and a lifelong commitment to learning.

## Conclusion

Our research highlights academic motivation as a crucial mediating factor, demonstrating that enhancing students’ digital skills and fostering academic motivation are vital for promoting lifelong learning behaviors. Additionally, the strong correlations among various dimensions of digital competence and academic motivation underscore the interconnected nature of these constructs. By recognizing the importance of digital competence and motivation, educational institutions can better support students in developing the skills necessary for successful lifelong learning in an increasingly digital world.

## Electronic supplementary material

Below is the link to the electronic supplementary material.

Supplementary Material 1

## Data Availability

The datasets and materials of the current study are available from the corresponding author on reasonable request.

## Abbreviations

AMS: Academic Motivation Scale
CFA: Confirmatory Factor Analysis

## References

[1] Mehrvarz M, Heidari E, Farrokhnia M, Noroozi O. The mediating role of digital informal learning in the relationship between students’ digital competence and academic performance. Comput Educ. 2021;167:104184. 10.1016/j.compedu.2021.104184. 10.1111/jcal.12553 PMC8250506 34230741

[2] Marawa’a A. E-Learning experiences among nursing students: A scoping review. Adv Med Educ Pract. 2024;369–79. 10.2147/AMEP.S453153. 10.2147/AMEP.S453153 PMC11075691 38715711

[3] Pan L, Haq Sul, Shi X, Nadeem M. The impact of digital competence and personal innovativeness on the learning behavior of students: exploring the moderating role of digitalization in higher education quality. Sage Open. 2024;14(3). 10.1177/21582440241265919.

[4] Tørris C, Meyer ME, Sandbekken IH, Halvorsrud H, Molin M. Nursing students’ perceived learning outcomes, motivation to learn and grade achieved in a digital blended learning course: A Norwegian cross-sectional study. Educ Sci. 2022;12(7):467. 10.3390/educsci12070467.

[5] El-Sayed BKM, El-Sayed AAI, Alsenany SA, Asal MGR. The Role of Artificial Intelligence Literacy and Innovation Mindset in Shaping Nursing Students’ Career and Talent Self-Efficacy. Nurse Educ Pract. 2024;104208. 10.1016/j.nepr.2024.104208. 10.1016/j.nepr.2024.104208 39637623

[6] Mainz A, Nitsche J, Weirauch V, Meister S. Measuring the digital competence of health professionals: scoping review. JMIR Med Educ. 2024;10(1):e55737. 10.2196/55737. 38551628 10.2196/55737 PMC11015375

[7] Tischendorf T, Hasseler M, Schaal T, Ruppert SN, Marchwacka M, Heitmann-Möller A, Schaffrin S. Developing digital competencies of nursing professionals in continuing education and training–a scoping review. Front Med. 2024;11:1358398. 10.3389/fmed.2024.1358398. 10.3389/fmed.2024.1358398 PMC11212473 38947234

[8] Stunden A, Ginige A, O’Reilly R, Sanagavarapu P, Heaton L, Jefferies D. Nursing students’ preparedness for the digitalized clinical environment in Australia: an integrative review. Nurse Educ Pract. 2024;103908. 10.1016/j.nepr.2024.103908. 10.1016/j.nepr.2024.103908 38335697

[9] Pajari J, Sormunen M, Salminen L, Elonen I, Pasanen M, Saaranen T. An instrument to assess the digital competence of nurse educators. Nurse Educ. 2024. 10.1097/nne.0000000000001637. 38723256 10.1097/NNE.0000000000001637

[10] Asal MGR, Alsenany SA, Elzohairy NW, El-Sayed AAI. The impact of digital competence on pedagogical innovation among nurse educators: the moderating role of artificial intelligence readiness. Nurse Educ Pract. 2025;104367. 10.1016/j.nepr.2025.104367. 10.1016/j.nepr.2025.104367 40209516

[11] Leonardsen ACL, Hardeland C, Hallgren J, Femdal I, Thapa DR, Helgesen AK, Gillsjö C. Nursing students’ attitudes towards the use of digital technology in the healthcare of older adults-a cross-sectional study in Norway and Sweden. BMC Nurs. 2023;22(1):428. 10.1186/s12912-023-01600-6. 37964266 10.1186/s12912-023-01600-6 PMC10644650

[12] Ibrahim RK, Aldawsari AN. Relationship between digital capabilities and academic performance: the mediating effect of self-efficacy. BMC Nurs. 2023;22(1):434. 10.1186/s12912-023-01593-2. 37978508 10.1186/s12912-023-01593-2 PMC10655374

[13] Garzón-Artacho E, Sola-Martínez T, Romero-Rodríguez J-M, Gómez-García G. Teachers’ perceptions of digital competence at the lifelong learning stage. Heliyon. 2021;7(7):e07513. 10.1016/j.heliyon.2021.e07513. 34401558 10.1016/j.heliyon.2021.e07513 PMC8353311

[14] El-Sayed AA, Abdelaliem SM. Application of Kano model for optimizing the training system among nursing internship students: a mixed-method Egyptian study. BMC Nurs. 2023;22(1):316. 10.1186/s12912-023-01485-5. 37710268 10.1186/s12912-023-01485-5 PMC10500916

[15] Tang SC, Tang LC. Exploring the impact of digital concept mapping methods on nurse students’ learning anxiety and learning motivation. Eval Program Plan. 2024;106:102466. 10.1016/j.evalprogplan.2024.102466. 10.1016/j.evalprogplan.2024.102466 39032440

[16] Nguyen MTH, Le MQ, Vo TM, Van Huynh L, Nguyen PV, Nguyen TH. Factors associated with academic motivation in nursing students: A cross-sectional study. Jurnal Kebidanan Dan Keperawatan Aisyiyah. 2023;19(1):1–14. 10.31101/jkk.3027.

[17] Seo YK, Kang CM, Kim KH, Jeong IS. Effects of gamification on academic motivation and confidence of undergraduate nursing students: A systematic review and meta-analysis. Nurse Educ Today. 2024;106388. 10.1016/j.nedt.2024.106388. 10.1016/j.nedt.2024.106388 39303410

[18] Amin SM, El-Sayed AA, Alsenany SA, Atta MH, Morsy OM, Asal MG. How clinical reasoning and Decision-Making competences influence the provision of empathic care among nursing students? Teaching and learning in nursing. 2025 Jan 25. 10.1016/j.teln.2025.01.005

[19] Rojas Eccoña P, Choque Cáceres AA, Berduzco-Torres N. Learning styles and digital competence in nursing students in hybrid academic modality. RIDE Revista Iberoamericana Para La Investigación Y El Desarrollo Educativo. 2023;14(27). 10.23913/ride.v14i27.1617.

[20] Collins GD. Developing nursing students’ digital capabilities to enhance public health. Prim Health Care. 2024;34(5). 10.7748/phc.2024.e1846.

[21] Zhao Y, Llorente AMP, Gómez MCS. Digital competence in higher education research: A systematic literature review. Comput Educ. 2021;168:104212. 10.1016/j.compedu.2021.104212. 36568577 10.1016/j.compedu.2021.104212 PMC9759745

[22] Haleem A, Javaid M, Qadri MA, Suman R. Understanding the role of digital technologies in education: A review. Sustainable Oper Computers. 2022;3:275–85. 10.1016/j.susoc.2022.05.004.

[23] Kwiatkowska W, Wiśniewska-Nogaj L. Digital skills and online collaborative learning: the study report. Electron J e-Learning. 2022;20(5):510–22. 10.34190/ejel.20.5.2412.

[24] Sarva E, Lastovska A, Olesika A, Zalite-Supe Z, Rubene Z, Abolina A. Development of university education field student digital competencies-overview of practice. INTED2024 Proc. 2024;173–80. 10.21125/inted.2024.0091.

[25] Chukwuedo SO, Mbagwu FO, Ogbuanya TC. Motivating academic engagement and lifelong learning among vocational and adult education students via self-direction in learning. Learn Motiv. 2021;74:101729. 10.1016/j.lmot.2021.101729.

[26] Şenyuva E, Kaya H. Do the lifelong learning tendencies of nursing students affect their attitudes toward E-Learning? Florence Nightingale J Nurs. 2022;30(3):259. 10.5152/FNJN.2022.21164. 36106808 10.5152/FNJN.2022.21164 PMC9623200

[27] Hamm J, Pi-Ming YEH. Factors affecting academic motivation in undergraduate nursing students: A scoping review. Nurse Educ Pract. 2024;104135. 10.1016/j.nepr.2024.104135. 10.1016/j.nepr.2024.104135 39270487

[28] Hwang Y, Oh J. The relationship between self-directed learning and problem-solving ability: the mediating role of academic self-efficacy and self-regulated learning among nursing students. Int J Environ Res Public Health. 2021;18(4):1738. 10.3390/ijerph18041738. 33670105 10.3390/ijerph18041738 PMC7916894

[29] Dang TD, Phan TT, Vu TNQ, La TD, Pham VK. Digital competence of lecturers and its impact on student learning value in higher education. Heliyon. 2024;10(17):e37318. 10.1016/j.heliyon.2024.e37318. 39296218 10.1016/j.heliyon.2024.e37318 PMC11408828

[30] Chen C, Shen Y, Zhu Y, Xiao F, Zhang J, Ni J. The effect of academic adaptability on learning burnout among college students: the mediating effect of self-esteem and the moderating effect of self-efficacy. Psychol Res Behav Manage. 2023;1615–29. 10.2147/PRBM.S408591. 10.2147/PRBM.S408591 PMC10164379 37163132

[31] El-Sayed AA, Goda SF, Elbialy GG. Threats of nursing productivity in the digital era: investigating the interplay between smartphone addiction and procrastination behavior among nurses. BMC Nurs. 2024;23(1):577. 10.1186/s12912-024-02218-y. 39164661 10.1186/s12912-024-02218-y PMC11337763

[32] Arbulú Pérez Vargas CG, Muro M, Pérez JP, Delgado JW, Carrasco E, Fernández DK, Cueva A, Acosta-Enriquez BG. The mediating role of digital skills and mobile self-efficacy in the stress and academic engagement of Peruvian university students in post-pandemic virtual environments. BMC Psychol. 2024;12(1):481. 10.1186/s40359-024-01982-5. 39256869 10.1186/s40359-024-01982-5 PMC11389404

[33] Martzoukou K, Luders ES, Mair J, Kostagiolas P, Johnson N, Work F, Fulton C. A cross-sectional study of discipline‐based self‐perceived digital literacy competencies of nursing students. J Adv Nurs. 2024;80(2):656–72. 10.1111/jan.15801. 37489586 10.1111/jan.15801

[34] Galindo-Domínguez H, Bezanilla MJ. Promoting time management and self-efficacy through digital competence in university students: A mediational model. Contemp Educational Technol. 2021;13(2):ep294. 10.30935/cedtech/9607.

[35] Marshall S, Blaj-Ward L, Dreamson N, Nyanjom J, Bertuol MT. The reshaping of higher education: technological impacts, pedagogical change, and future projections. High Educ Res Dev. 2024;43(4):521–41. 10.1080/07294360.2024.2329393.

[36] Ali HFM, Mousa MAEG, Hussein Ramadan Atta MHR, et al. Exploring the association between internet addiction and time management among undergraduate nursing students. BMC Nurs. 2024;23:632. 10.1186/s12912-024-02273-5. 39256720 10.1186/s12912-024-02273-5 PMC11389558

[37] Caena F, Redecker C. Aligning teacher competence frameworks to 21st century challenges: the case for the European digital competence framework for educators (Digcompedu). Eur J Educ. 2019;54(3):356–69. 10.1111/ejed.12345.

[38] Wigfield A, Eccles JS. The relevance of situated expectancy-value theory to understanding motivation and emotion in different contexts. In: Hagenauer G, Lazarides R, Järvenoja H, editors. Motivation and emotion in learning and teaching across educational contexts: theoretical and methodological perspectives and empirical insights. Abingdon: Routledge; 2023. p. 3–18.

[39] Eccles JS, Wigfield A. The development, testing, and refinement of Eccles, Wigfield, and colleagues’ situated Expectancy-Value model of achievement performance and choice. Educ Psychol Rev. 2024;36:51. 10.1007/s10648-024-09888-9.

[40] Burnett N. The Delors report: a guide towards education for all. Eur J Educ. 2008;43(2):181–7. 10.1111/j.1465-3435.2008.00347.x.

[41] Zimmerman BJ. Becoming a self-regulated learner: an overview. Theory into Pract. 2002;41(2):64–70. 10.1207/s15430421tip4102_2.

[42] Zimmerman BJ, Schunk DH. (2012). Motivation: An essential dimension of self-regulated learning. In Motivation and self-regulated learning (pp. 1–30). Routledge. 10.4324/9780203831076

[43] Faul F, Erdfelder E, Lang AG, Buchner A. G* power 3: A flexible statistical power analysis program for the social, behavioral, and biomedical sciences. Behav Res Methods. 2007;39(2):175–91. 10.3758/BF03193146. 17695343 10.3758/bf03193146

[44] Tzafilkou K, Perifanou M, Economides AA. Development and validation of students’ digital competence scale (SDiCoS). Int J Educational Technol High Educ. 2022;19(1):30. 10.1186/s41239-022-00330-0. 10.1186/s41239-022-00330-0 PMC9107949 35602658

[45] Wielkiewicz RM, Meuwissen AS. A lifelong learning scale for research and evaluation of teaching and curricular effectiveness. Teach Psychol. 2014;41(3):220–7. 10.1177/0098628314537971.

[46] Vallerand RJ, Pelletier LG, Blais MR, Briere NM, Senecal C, Vallieres EF. The academic motivation scale: A measure of intrinsic, extrinsic, and motivation in education. Educ Psychol Meas. 1992;52(4):1003–17. 10.1177/0013164492052004025.

[47] Fatima M, Ahmed S, Jokhio PB, Rind A, Haroon RM, Malhi LD. Evaluating nursing students’ academic motivation through academic motivational scale: A Cross-Sectional study of two colleges. Language. 2021;59:44–7. 10.37184/lnjpc.2707-3521.3.8.

[48] Rushdan EE, Atta MHR, Nashwan AJ, Zoromba M, Ali HFM. (2024). Comparative analysis of engagement and academic self-concept among nursing students: Differences in study modalities. Nursing Forum, 2024, Article 6621905. 10.1155/2024/6621905

[49] Atta MH, Hammad H, Elzohairy NW. The role of empathy in the relationship between emotional support and caring behavior towards patients among intern nursing students. BMC Nurs. 2024;23. 10.1186/s12912-024-02074-w. 10.1186/s12912-024-02074-w PMC11212155 38943109

[50] Koskinen I, Stolt M, Widmer CT, Pernica K, Dütthorn N, Groddeck L,... Virtanen H. Methodological approaches and competence areas of nursing students in virtual reality simulation research–A scoping review. Nurse Education Today. 2024;133:106033. 10.1016/j.nedt.2023.106033. 10.1016/j.nedt.2023.106033 37988799

[51] Hoe SL. Issues and procedures in adopting structural equation modeling technique. J Quant Methods. 2008;3(1):76.

[52] Selim A, Newby C, Almutairy A, Aldossari A, Alkabba F, Arabi S, Zoromba MA, Atta MHR, Ibrahim N. Enhancing warning signs of mental health literacy: evaluating a digital base intervention for health profession students. Arch Psychiatr Nurs. 2025;54:91–101. 39955149 10.1016/j.apnu.2025.01.005

