# Supplementary material for: Nursing education in the digital era: the role of digital competence in enhancing academic motivation and lifelong learning among nursing students
Source: BMC Nurs. 2025 May 21;24:571. doi: 10.1186/s12912-025-03199-2 (PMC12093728; doi:10.1186/s12912-025-03199-2)
Supplement: Supplementary file 1 — Supplementary Material 1 [file 12912_2025_3199_MOESM1_ESM.docx]

**Table 1S: Distribution of the study participants according to their characteristics (n=500)**

| **Variable** | **Category** | **Frequency** | **Percent** |
| --- | --- | --- | --- |
| Age | >20 | 6 | 1.2 |
|  | 20- | 315 | 63.0 |
|  | 22+ | 179 | 35.8 |
| Gender | Male | 229 | 45.8 |
|  | Female | 271 | 54.2 |
| Occupation | Working | 111 | 22.2 |
|  | Not working | 389 | 77.8 |
| Residence | Rural | 366 | 73.2 |
|  | Urban | 134 | 26.8 |
| Family type | Nuclear | 258 | 51.6 |
|  | Extended | 242 | 48.4 |
| Family income | Enough | 300 | 60.0 |
|  | Enough & save | 136 | 27.2 |
|  | Not-enough | 64 | 12.8 |
| Mother's educational level | Illiterate | 26 | 5.2 |
|  | Basic education | 97 | 19.4 |
|  | Secondary education | 237 | 47.4 |
|  | University & above | 140 | 28.0 |
| Father's Educational Level | Illiterate | 28 | 5.6 |
|  | Basic education | 94 | 18.8 |
|  | Secondary education | 208 | 41.6 |
|  | University & above | 170 | 34.0 |
| Technology Access | Yes | 289 | 57.8 |
|  | No | 211 | 42.2 |
| Internet Access | Home | 225 | 45.0 |
|  | University/college | 105 | 21.0 |
|  | No access | 39 | 7.8 |
|  | Mobile data | 131 | 26.2 |
| Teaching method | Traditional lectures | 145 | 29.0 |
|  | Hybrid learning | 149 | 29.8 |
|  | E-learning | 206 | 41.2 |
| Clinical Teaching method | Simulation-based learning | 215 | 43.0 |
|  | Field trip | 108 | 21.6 |
|  | Case-based learning | 23 | 4.6 |
|  | Problem-based learning | 154 | 30.8 |
| Experience with online learning platforms | Beginner | 160 | 32.0 |
|  | Intermediate | 128 | 25.6 |
|  | Advanced | 212 | 42.4 |
| Experience with digital tools | None | 217 | 43.4 |
|  | Less than one year | 102 | 20.4 |
|  | 1-3 years | 39 | 7.8 |
|  | More than three years | 142 | 28.4 |
|  | Total | 500 | 100.0 |
